# Philadelphia‐like acute lymphoblastic leukemia: Characterization in a pediatric cohort in a referral center in Colombia

**DOI:** 10.1002/cnr2.1587

**Published:** 2021-11-17

**Authors:** Adriana Linares Ballesteros, Luz Karime Yunis, Johnny García, Nelson Aponte, Jessica Flechas, Cindy Martinez, Gloria Uribe, Edna Quintero, Angela Díaz, Carlos Pardo, Isabel Cristina Sarmiento, Agustin Contreras, Juan Jose Yunis

**Affiliations:** ^1^ Pediatric Hematology/Oncology Unit HOMI Fundación Hospital Pediátrico la Misericordia Bogotá Colombia; ^2^ Grupo Oncohematología Pediátrica Universidad Nacional de Colombia Bogotá Colombia; ^3^ Servicios Médicos Yunis Turbay y Cía S.A.S. Instituto de Genética Bogotá Colombia; ^4^ Grupo de Patología Molecular Universidad Nacional de Colombia Bogotá Colombia; ^5^ Pediatric Pathology Unit HOMI Fundación Hospital Pediatrico la Misericordia Bogotá Colombia; ^6^ Departamento de Patología, Facultad de Medicina e Instituto de Genética Universidad Nacional de Colombia Bogotá Colombia

**Keywords:** acute lymphoblastic leukemia, *CDKN2A/B*, children, Colombia, *CRLF2*, *ETV6*, Hispanic, *IKZF1*, *PAX5*, Ph‐like

## Abstract

**Background:**

Philadelphia‐like (Ph‐like) acute lymphoblastic leukemia (ALL) is a subtype of pediatric leukemia with high risk factors and poor outcome. There are few reports of its prevalence in Latin America.

**Aim:**

This study evaluated the frequency and clinical and biological characteristics of Ph‐like ALL in a pediatric cancer center in Colombia.

**Methods:**

The Ph‐like genetic profile was analyzed by a low‐density array (LDA). Samples from patients with Ph‐like ALL were analyzed by fluorescent in situ hybridization for cytokine receptor like factor 2 (*CRLF2)* and ABL proto‐oncogene 1, non‐receptor tyrosine kinase (*ABL1)* rearrangements. Copy number variations were assessed by multiplex ligation probe amplification.

**Results:**

Data from 121 patients were analyzed. Fifteen patients (12.4%) had Ph‐like ALL, and these patients had significantly higher leukocyte counts at diagnosis and higher levels of minimal residual disease on days 15 and 33 of induction than patients without the Ph‐like subtype. There were no significant differences in sex, age, or response to prednisone at day 8 between the two groups. *CRLF2* rearrangements were identified in eight patients, and *ABL1* rearrangements were identified in two patients. Other genetic alterations alone or in combination were identified in 77% of patients, including deletions in cyclin dependent kinase inhibitor 2 A/B (46.2%), IKAROS family zinc finger 1 (38.3%), and paired box 5 (30.8%).

**Conclusions:**

Ph‐like ALL had a 12.4% prevalence in our cohort of patients with pediatric ALL. The identification of this group of patients has importance for risk stratification and future targeted therapy.

## 1 INTRODUCTION

Acute lymphoblastic leukemia (ALL) is the most common pediatric neoplasm, in developed countries, long‐term survival close to 85% has been achieved with treatment strategies based on disease‐risk stratification.^1^, ^2^ Genetic alterations confer distinctive biological and clinical behaviors, which influence the response to treatment and are considered in the risk classification of modern protocols.

More than a decade ago, two academic groups, COG/St. Jude in the United States and DCOG in the Netherlands, simultaneously described a B‐ALL subtype with a gene expression profile similar to the one already recognized for BCR activator of RhoGEF and GTPase (*BCR)‐*ABL proto‐oncogene 1, non‐receptor tyrosine kinase (*ABL1)* but without the BCR‐ABL1 fusion protein, which is known as Philadelphia‐like (Ph‐like) or *BCR‐ABL1*‐like.^3^, ^4^ The Ph‐like subtype was recognized as a provisional entity in the last acute leukemia classification of the World Health Organization in 2016.^5^ This subtype is characterized by multiple genetic alterations in cytokine receptors and kinase signaling pathways. More than 70 alterations have been described, including translocations, cryptic rearrangements, single nucleotide variants, and copy number variations (CNVs).^6^ Alterations are also detected in the transcription factors involved in B lymphocyte development and maturation, including IKAROS family zinc finger 1 (*IKZF1)*, EBF transcription factor 1 (*EBF1)*, ETS variant transcription factor 6 (*ETV6)*, ETS transcription factor ERG, transcription factor 3 (*TCF3*), and paired box 5 (*PAX5)*, in 86% of the cases and with a frequency very similar to what is observed in Philadelphia‐positive B‐ALL cases.^7^ However, there is currently no consensus on how to diagnose and characterize the Ph‐like expression profile in patients with B‐ALL.^6^, ^8^

The most frequent genetic alterations in patients with Ph‐like ALL are rearrangements of cytokine receptor like factor 2 (*CRLF2)*,^9^ which are strongly associated with adverse clinical and treatment response factors and a higher risk of relapse with conventional therapy. Such rearrangements are found more frequently in the Hispanic population^10^ within cohorts of patients treated in North America. Unfortunately, there are little data on the frequency of Ph‐like B‐ALL in the Hispanic pediatric population outside the United States.^11^

The aim of this study was to identify the frequency of Ph‐like B‐ALL and examine its genomic and clinical characteristics in a cohort of patients under 18 years of age from a referral center in Colombia.

## 2 METHODS

This was a prospective, descriptive study, nested in a cohort of patients between 1 and 18 years of age with a confirmed diagnosis of B‐ALL from July 1, 2018 to August 1, 2020. Participants were selected at convenience from patients with a de novo confirmed diagnosis of B‐precursor ALL at the HOMI Fundación Hospital Pediátrico La Misericordia who received the ALL‐IC 2009 treatment protocol. Of 137 admitted patients, 10 were excluded due to lack of sample to perform Ph‐like expression profiling and 6 were excluded because they were BCR‐ABL1‐positive. Therefore, 121 patients were included in the analysis.

### 2.1 Biopsy and bone marrow aspiration procedure

Once consent was obtained and information formats were reviewed and completed, a bone marrow aspirate sample was taken to process flow cytometry (FC) to identify recurrent translocations, including *t*(4; 11), *t*(12; 21), and *t*(9; 22). In addition, a sample was taken for DNA and RNA extraction, conventional and molecular cytogenetics tests, and CNV analysis by multiplex ligation probe amplification (MLPA). Samples in which recurrent translocations were not found underwent low density array (LDA) analysis for the Ph‐like expression profile. Those with a positive LDA test underwent fluorescence in situ hybridization (FISH) for *CRLF2*, *ABL1*, and *ABL2*.

### 2.2 Immunophenotype analysis by FC

The Euroflow Screening Panel was used to analyze the immunophenotype of different maturation stages of neoplastic B cells in the bone marrow samples, with a combination of eight different fluorescent markers that allow the identification and phenotypic characterization of B lineage acute leukemias.^12^

### 2.3 DNA extraction

DNA was extracted from 200 μl of bone marrow using the QIAamp DNA Blood Mini Kit (Qiagen, Hilden, Germany) following the manufacturer's specifications. The DNA purity quantification and verification were performed using a NanoDrop 2000 spectrophotometer. The DNA obtained was stored at −20°C until use.

### 2.4 RNA extraction

RNA was isolated from bone marrow samples in EDTA using the Quick‐RNA MiniPrep Plus kit (ZYMO RESEARCH, Irvine, CA, USA) following the manufacturer's recommendations. The extracted RNA was converted into cDNA using the High‐Capacity cDNA Reverse Transcription kit (Applied Biosystems, San Francisco, CA, USA) following the manufacturer's recommendations. The DNA purity quantification and verification were assessed using a NanoDrop 2000. The RNA was stored at −80°C, and the cDNA was stored at −20°C until use.

### 2.5 Cytogenetics

Cell culture was performed to obtain metaphases for the chromosomal study with G and Q bands according to standardized protocols.^13^ Chromosome visualization was performed using GenASIs (Applied Spectral Imaging, Carlsbad, CA, USA). At least 25 metaphases per sample were analyzed, and the nomenclature was described according to the recommendations of the International System for Human Cytogenomic Nomenclature (ISCN) 2020.

### 2.6 Fluorescence in situ hybridization

FISH was performed to detect the following recurrent rearrangements: *t*(12; 21) (TEL‐AML1 [ETV6‐RUNX1] Translocation, Dual Fusion Probe, Cytocell, Cambridge, UK), *t*(4; 11) (MLL [KMT2A]/AFF1 Translocation, Dual Fusion Probe, Cytocell), and *t*(9; 22) (BCR‐ABL Translocation, Dual Fusion Probe, Cytocell). Likewise, in samples with an LDA‐positive Ph‐like expression profile, FISH was performed to assess *CRLF2* (CRLF2 Breakapart FISH probe, Cytocell), *ABL1* (ABL1 Breakapart FISH probe, Cytocell), and *ABL2* (ABL2 Breakapart FISH probe, Cytocell) translocations. At least 100 nuclei per study were analyzed, and the interpretation was performed by two independent observers using GenASIs. The nomenclature was described according to the recommendations of the ISCN 2020.

### 2.7 Multiplex ligation probe amplification

All patients underwent MLPA analysis to detect copy number alterations with the SALSA MLPA P335 ALL kit. The *IKZF1* probe mix (MRC Holland, Amsterdam, The Netherlands), was used following the manufacturer's recommendations to detect CNVs of *IKZF1*, *EBF1*, *CDKN2A/B*, *PAX5*, *ETV6*, BTG anti‐proliferation factor 1 (*BTG1*), RB transcriptional corepressor 1 (*RB1*), and the PAR1 region.

### 2.8 LDA expression profiling for Ph‐like ALL

Expression analysis was performed using a TaqMan Gene Expression assay (Applied Biosystems), TaqMan® Gene Expression Master Mix, and TaqMan® probes on an ABI7500 Real‐Time PCR system (Applied Biosystems). The expressions of bone morphogenetic protein receptor type 1B (*BMPR1B*); adhesion G protein‐coupled receptor F1 (*ADGRF1*); *CRLF2*; mucin 4 cell surface associated (*MUC4*); neurexin 3 (*NRXN3*); carbonic anhydrase 6 (*CA6*); joining chain of multimeric IgA and IgM (*JCHAIN*); and spermatogenesis associated serine rich 2 like (*SPATSL2*) were assessed,^14^ with some modifications, we used as a housekeeping gene glucose‐6‐phosphate dehydrogenase (*G6PD)*, to normalize the data. Also, each sample was processed in triplicate, and the gene expression was obtained using the 2^−ΔΔCt^ method. The LDA expression profile was considered positive when four or more genes (>0.5) had expression levels higher than log_e_10 > 2.3 compared to the control gene. Additionally, samples with individual overexpression of *CRLF2* in the LDA profile were analyzed by *CRLF2* FISH.

### 2.9 Statistical analysis

Statistical analyses of quantitative variables were carried out with measures of central tendency and dispersion, means and standard deviations, or medians and ranges, according to the distribution after analysis with normality tests (Kolmogorov–Smirnov or Shapiro–Wilk) to establish the behavior of the data as parametric or non‐parametric. Medians were compared using the Mann–Whitney *U* test for independent non‐parametric samples. Qualitative variables were analyzed with Pearson's Chi‐square test and Fisher's exact test. Statistical analysis was performed using the Statistical Package for Social Sciences (SPSS) for Windows, version 25.0. A *p <* .05 was considered significant.

For bias control, all diagnosed children entered the study, thus minimizing selection bias. Validated techniques were used, as well as positive and negative controls that accompanied the processing of the samples. Information was double‐checked when entered into the database for quality control.

## 3 RESULTS

One hundred and thirty‐seven patients were included in the study. Six patients with *BCR‐ABL1* translocations were excluded, as were 10 patients in whom we were unable to perform LDA due to the poor quality of the RNA obtained. For the final analysis, 121 patients were included. In 15 patients (12.4%), a positive gene expression profile for Ph‐like B‐ALL was observed (the Ph‐like group). The non‐Ph‐like group comprised 94 patients with a negative LDA Ph‐like expression profile and 12 patients with recurrent translocations (*n* = 106).

The median age for the entire cohort was 5 years (range 1–17 years), with the Ph‐like group having a median age of 9 years (range 1–17 years) and the non‐Ph‐like group having a median age of 5 years (range 1–17 years). This difference was not statistically significant (*p* = .250). The median leukocyte counts at diagnosis in the Ph‐like and non‐Ph‐like groups were 62.8 × 10^9^/L (range 12.9–447.2 × 10^9^/L) and 7.08 × 10^9^/L (range 0.5–345.1 × 10^9^/L), respectively. This difference was statistically significant (*p* < .001). The most frequent genetic alteration found was hyperdiploidy (*n* = 43, 35.5%).

Table 1 shows the biological and clinical characteristics of the patients according to the presence of the Ph‐like profile. More patients in the Ph‐like group had minimal residual disease (MRD) at day 15 and at day 33 (end of induction) than the non‐Ph‐like group (60 vs. 17.9%). Three patients in the Ph‐like group were originally considered to be high‐risk, considering the initial factors (age, leukocyte count, and prednisone response on day 8). With the MRD evaluation on days 15 and 33, five more patients were added to the high‐risk group at the end of induction. Two patients in each group received hematopoietic stem cell transplantation; three were alive at the end of the study period, and one patient in the Ph‐like group died after transplantation. One and six patients in the Ph‐like and non‐Ph‐like groups had an early relapse, all within 36 months of diagnosis.

**TABLE 1:** Demographic data of the two groups of patients

Patients characteristics	Ph‐like (+) *n* = 15	Ph‐like (−) *n* = 106
Gender (M:F)	7/8	55/51
Age at diagnosis (years) ≥ 10 years	4	71
<10 years	11	35
WBC at diagnosis		
<20 x 10^9^/L	6	79
20 a 100 x 10^9^/L	3	23
>100 x 10^9^/L	6	4
CNS involvement	2	9
Day 8 prednisone response		
<1000 blasts in peripheral blood	12	86
>1000 blasts in peripheral blood	3	20
Day 15 MRD		
<0,1%	2	30
0.1–10%	6	53
>10%	7	21
Day 33 MRD		
<0.01%	6	78
≥0.01%	9	19
Failure at end of induction (>5% blasts morphological on bone marrow)	2	2
Risk classification at end of induction		
Standard	1	14
Intermediate	6	62
High	8	30

Abbreviations: CNS, central nervous system; MRD, minimal residual disease (measured by flow cytometry); WBC, white blood count.

The clinical data and treatment response of the Ph‐like group are shown in Table 2. The Phi‐like expression profile was identified in 15/121 patients (12.4%). *CRLF2* rearrangements were identified in 8/15 patients (53.3%), 4 with *IGH‐CRLF2* and 4 with *P2RY8‐CRLF2*, and *ABL1* rearrangements were found in 2/15 patients (13.3%). The RAS signaling pathway was not evaluated. Half of the patients with *CRLF2* rearrangements also harbored *IKZF1* deletions. Other associated genetic alterations were analyzed by MLPA in 13 of the 15 patients in the Ph‐like group. Genetic alterations were detected in 10 of 13 patients (77%), either alone or in combination. The most common mutations were *CDKN2A/B* deletions (*n* = 6, 46.2%), *IKZF1* deletions (*n* = 5, 38.3%), and *PAX5* deletions (*n* = 4, 30.8%). Two patients had other alterations (15.4%). Three patients (23.1%) had no alterations. Of the 15 patients in the Ph‐like group, three had hyperdiploidy (two had rearrangements in *CRLF2*), and two had intrachromosomal amplification of chromosome 21 (iAMP21).

**TABLE 2:** Clinical characteristics and initial treatment response of the 15 patients with Ph‐like ALL

Age (years)	Gender	WBC(x 10^9^/L)	Prednisone response at day 8 (good‐poor)	Count blasts by FCM at day 15 (%)	Count blasts by FCM at day 33 (%)	*CRLF2* rearrangements	*ABL1* rearrangements	Copy number variations	Relapse Yes /No	Status dead /Alive
3	F	9.44	Good	7.66	0	Negative	Negative	No CNV	No	Alive
10	F	388	Poor	50.5	2.04	*IGH‐CRLF2*	Negative	*IKZF1, CDKN2A/B, BTG1* DELETIONS	No	Dead
11	M	151.1	Poor	61.1	0	*IGH‐CRLF2*	Negative	*IKZKF1, RB1, PAX5* DELETIONS	No	Alive
9	M	447.2	Poor	86.59	7.68	Negative	Positive	*IKZK1, CDKNDA/B, ETV6, BTG1* DELETIONS	Yes	Dead
1	M	118.6	Good	10.7	0.06	*P2RY8‐CRLF2*	Negative	*PAX5* DELETION	No	Alive
9	M	9.9	Good	7.96	0.01	*P2RY8‐CRLF2*	Negative	Not performed	No	Alive
9	F	9.2	Good	54.3	3.44	Negative	Positive	*RB1* DELETION	No	Dead
3	F	7.3	Good	0.07	0.02	*P2RY8‐CRLF2*	Negative	*ILR3A, P2RY8, CSF22RA* DELETIONS	No	Alive
17	M	79.7	Good	4.76	0	*IGH‐CRLF2*	Negative	*IKZF1, CDKN2A/B, PAX5, ETV6* DELETIONS	No	Alive
8	M	1.2	Good	0.96	0	*P2RY8‐CRLF2*	Negative	No CNV	No	Alive
16	F	53.8	Good	0	0	Negative	Negative	*CDKN2A/B* DELETIONS	No	Alive
6	M	14.4	Good	7.46	0.07	Negative	Negative	No CNV	No	Dead
9	F	171.8	Good	79.44	33.41	Negative	Negative	No CNV	No	Alive
9	M	255.9	Good	5.7	0	*IGH‐CRLF2*	Negative	*IKZF1, CDKN2B* DELETIONS	No	Alive
3	F	6.8	Good	15.25	0.13	Negative	Negative	*JAK2, CDKN2A/B, PAX5* DELETIONS	No	Alive

Abbreviations: CNV, copy number variations; F, female; M, male.

**FIGURE 1:**
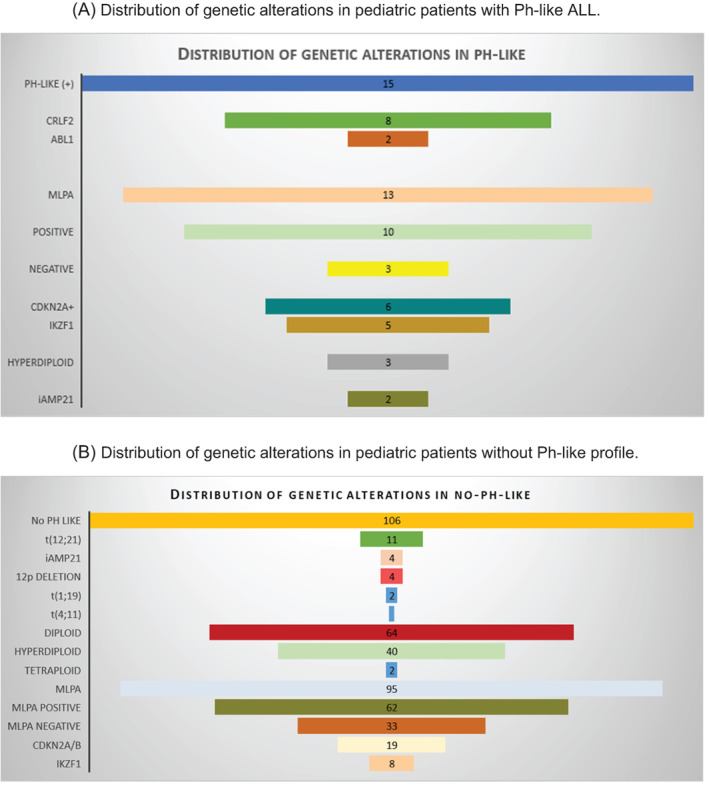
(A) Distribution of genetic alterations in pediatric patients with Ph‐like ALL. (B) Distribution of genetic alterations in pediatric patients without Ph‐like profile

In the non‐Ph‐like group, *t*(12; 21) translocation was identified in 11 patients, iAMP21 amplification in four patients, 12p deletion in four cases, *t*(1; 19) translocation in two patients, and *t*(4; 11) translocation in one patient. Sixty‐four patients had a diploid karyotype, 40 had hyperdiploidy, and two had a tetraploid karyotype. The cytogenetic and molecular findings of the Ph‐like and non‐Ph‐like groups are shown in Figure 1. MLPA studies were performed in 95 patients; 62 (65.3%) had CNVs (alone or in combination with other genetic abnormalities), 19 (20.0%) had *CDKN2A/B* deletions (20%), 8 (8.4%) had *IKZK1* deletions, 9 (9.5%) had *ETV6* deletions, and 6 (6.3%) had isolated deletion of *PAX5*.

Overexpression of *CRLF2* without a positive LDA for the Ph‐like expression profile was detected in six patients (5.7%) with negative FISH results for *IGH‐CRLF2* and *P2RY8‐CRLF2* rearrangements. The median age of these patients was 4 years (range 2–9 years), 5/6 patients had a leukocyte count less than 10 × 10^9^/L, and none had a leukocyte count higher than 50 × 10^9^/L. Five had hyperdiploidy, none had translocations associated with increased risk, such as *t*(4; 11) or *t*(12; 21), all had a good response to prednisone at day 8, and all had MRD ≤0.01 at day 33. Although one patient died on induction, no other patients experienced other adverse events, including recurrence, at the time of this evaluation.

When comparing the group of patients with and without Ph‐like B‐ALL, differences were found in demographic and clinical variables (Table 3).

**TABLE 3:** Association of demographic and clinical variables with Ph‐like B‐ALL and non‐Ph‐like B‐ALL

Variable	Ph‐like	No Ph‐like	OR	IC 95%	*p*
Gender (M:F)	8/7	55/51	1.06	0.36–3.13	*0.916*
Leukocytes at diagnosis >20 x 10^9^/L	9/15 (60%)	27/106 (25%)	4.38	1.43–13.47	***0.009***
Leukocytes at diagnosis >50 x 10^9^/L	9/15 (60%)	14/106	18.37	5.21–64.75	***<0.001***
Leukocytes at diagnosis >100 x 10^9^/L	6/15 (40%)	4/106 (3,7%)	17	4.04–71.54	***<0.001***
Day 8 prednisone response >1000 absolute blasts	3/15 (20%)	20/106 (19%)	1.07	0.27–4.17	*0.575*
Day 15 MRD >1%	12/15 (80%)	50/104 (48%)	6.75	1.45–31.43	***0.005***
Day 15 MRD >10%	7/15 (47%)	21/104 (20%)	3.45	1.12–10.61	***0.032***
Day 33 MRD >0,01%	9/15 (60%)	17/97 (17%)	6.15	1.95–19.41	***0.002***
Day 33 Bone marrow morphologic remission	13/15 (87%)	95/97 (98%)	7.3	0.96–57.41	*0.086*
*IKZF1* positive status	5/13 (38%)	8/95 (8,4%)	6.79	1.79–25.73	***0.009***

*Note*: Bold values represent a Ph‐like: philadelphia like, No Ph‐like: no philadelphia like, OR: odds ratio, CI 95%: confidence interval 95%, *p < 0,002*.

Abbreviation: MRD, minimal residual disease measured by flow cytometry.

The follow‐up time of this cohort was 10–34 months. The mortality was similar between the groups (Ph‐like: 26% and non‐Ph‐like: 18.8%). Most of the deaths in both groups were treatment‐related, although one patient in each group died due to refractory relapse. At the time of this evaluation, six patients had relapsed in the non‐Ph‐like group, whereas no living patients in the Ph‐like group had relapsed.

## 4 DISCUSSION

In this study, we examined the frequency of Ph‐like B‐ALL and assessed its genomic and clinical characteristics in a cohort of patients under 18 years of age from a referral center in Colombia. A Ph‐like profile frequency of 12.4% was found in our cohort. This frequency is similar to that reported by other authors (between 10 and 19%).^7^, ^15^, ^16^ It is important to note that our patients are a Latino/Hispanic population outside of North America; there is very little published information on the frequency of this variant of ALL in children in Latin America. Perez‐Vera et al. published a summary reporting 53% of this subtype in two institutions in Mexico this is high compared with our findings, however their diagnostic methodology to consider a result positive was different from that used in this study.^11^ The clinical characteristics found in the Ph‐like group were similar to those found in other studies in children, such as higher white blood cell counts^17^, ^18^ and a higher frequency of failure of remission at the end of induction.^17^, ^18^, ^19^ No differences were found by sex, unlike the predominance in males found by other authors.^19^

MRD at the end of induction has become essential in many protocols to define risk and intensification of therapy; therefore, it is important to identify this subgroup of patients at an early point in treatment. In our study, a statistically significant difference was found in MRD at the end of induction between the Ph‐like and non‐Ph‐like groups (60 vs. 17% *p* = .002). Other authors have also reported this association in children with Ph‐like ALL.^18^, ^19^ In 20% of patients with Ph‐like ALL, the risk classification was modified to a higher classification when considering the MRD at the end of induction, but hematopoietic transplantation is not standard in our institutional protocol when patients have MRD positive at the end of induction, so patients with Ph‐like profile and MRD positive at the end of induction followed our protocol without transplantation in first remission .

*CRLF2* rearrangements were found in 53.3% of the patients with the Ph‐like gene expression profile, with the same proportion of patients having *IGH‐CRLF2* and *P2RY8‐CRLF2* rearrangements. These frequencies are variable in pediatric patients. There are reports of similar frequencies for both of these rearrangements,^18^ although some authors report a predominance of the *IGH*‐*CRLF2* rearrangement. Studies in adults with B‐ALL also report a higher frequency of *CRLF2* rearrangements in patients with a Ph‐like expression profile.^20^ Other genomic alterations, such as deletions of *IKZF1*, were observed in both groups, albeit less frequently. Deletions of *IKZF1* were observed in 38.6% of patients in the Ph‐like group, which is similar to the findings of Roberts et al.^19^ but lower than that reported in other pediatric cohorts (60–80%).^12^, ^21^ This result may be due to different sample sizes or patient populations, since some examined a selected high‐risk population. We found a small number of patients with *CRLF2* overexpression without an LDA‐positive Ph‐like expression profile. These patients did not harbor *CRLF2* rearrangements and did not have adverse prognostic factors, as has been described by other authors.^22^ Hyperdiploidy in patients with Ph‐like ALL is not a consistent finding; Van Der Veer et al they did not find hyperdiploidy in a group of patients ALL Ph‐like.^23^ On the other hand, Jain et al. that included 68% of Hispanic population living in United States, found hyperdiploidy in 20% of patients with Ph‐like profile, a similar proportion found in our study.^24^ Related to iAMP21, this alteration could be found in patients with Ph‐like profile. Schwab and Harrison reported that near 60% of iAMP21‐ALL patients had mutations in genes within the RAS signaling pathway, this pathway is part of the Ph‐like phenotype.^25^

Both groups had a similar proportion of high‐risk patients at the initial risk assessment (20 vs. 19%). In the Ph‐like group, the proportion of high‐risk patients increased to 53.5% at the end of induction, after taking the MRD into consideration. This finding is different from that of the study by Roberts et al,^19^ which included all patients from a cohort of children with ALL, in which no patients in the Ph‐like group were initially categorized as high‐risk. However, at the end of induction, 27% of patients in the Ph‐like group switched to the high‐risk group due to MRD. Today MRD is part of risk classification of ALL protocols in children.

Difficulties in diagnosing the Ph‐like expression profile persist, more than a decade after its identification. There is heterogeneity in the methods used to characterize the Ph‐like profile, and there is no consensus on the methodology used to identify this B‐ALL subtype. Other groups have reported difficulties in processing samples. The GIMEMA group analysis of samples from a cohort of adult ALL patients were retrospectively searched for the Ph‐like expression profile, and 16.2% of patients could not be analyzed due to RNA unavailability.^8^ It should be address in the near future a consensus for the diagnosis of Ph‐like ALL.

Identifying this B‐ALL subtype may provide an opportunity to understand why some *BCR‐ABL1*‐negative patients without findings of poor prognosis have persistent MRD at the end of induction and require treatment intensification. The current diagnostic strategies are not uniform, and many may not be feasible in countries with limited resources. It is necessary to design straightforward methodologies that can be easily implemented to identify this subgroup of patients.^26^

The best therapeutic strategy for this group of patients is still being examined, motivated by the impact of the revolutionary role of tyrosine kinase inhibitors such as imatinib in improving the outcomes of patients with Philadelphia‐positive B‐ALL. Several collaborative groups are currently conducting randomized clinical trials, whose results will be known over the next few years.^17^, ^24^ Among the questions to be resolved in this specific B‐ALL subtype is the possibility of using targeted therapy associated with conventional chemotherapy, which allows improving current results without increasing toxicity and eliminating the need for consolidation with HPT.^27^

In conclusion, the frequency of the Ph‐like ALL subtype in our cohort (12.4%) was similar to that described in cohorts in North America. This subtype was associated with poor prognostic factors, as previously identified. Additionally, 77% of these patients had gene deletions, such as *IKZF1* or *CDKN2A/B*. We should identify factors associated with a greater probability of having the Ph‐like expression profile in order to standardize methods to identify this B‐ALL variant in countries with limited resources. Identifying B‐ALL variants that require intensification of treatment, especially due to positive MRD at the end of induction, and which in the future may be susceptible to targeted therapies may improve the chances of cure for this subgroup of patients.

## CONFLICT OF INTEREST

The authors declare no conflict of interest.

*Conceptualization, Funding Acquisition, Investigation, Resources, Supervision, Writing—Original Draft, Writing—Review and Editing*, A.L.; *Conceptualization, Investigation, Methodology, Resources, Validation, Writing – Original Draft*, L.K.Y.; *Investigation, Project Administration, Writing—Original Draft, Writing—Review And Editing*, J.G.; *Data Curation, Formal Analysis, Software*, N.A.; *Data Curation, Investigation, Supervision*, J.F.; *Data Curation, Investigation, Project Administration, Supervision, Validation*, C.M.; *Formal Analysis, Methodology, Resources, Writing—Review and Editing*, G.U.; *Formal Analysis, Methodology, Resources, Writing—Review and Editing*, E.Q.; *Methodology, Resources, Validation*, A.D.; *Formal Analysis, Investigation, Writing—Review and Editing*, C.P.; *Formal Analysis, Investigation, Writing—Review and Editing*, I.S.; *Conceptualization, Funding Acquisition, Project Administration, Supervision, Writing—Review and Editing*, A.C.; *Conceptualization, Formal Analysis, Methodology, Resources, Writing – Original Draft, Writing—Review and Editing*, J.J.Y.

## ETHICAL STATEMENT

Informed consent was obtained from each patient or guardian. This protocol was approved by the institutional ethics committee of our institution (EC number 010 April 2019).

## Data Availability

The data that support the findings of this study are available from the corresponding author upon reasonable request.
